# Quality assessment of reporting of randomization, allocation concealment, and blinding in traditional chinese medicine RCTs: A review of 3159 RCTs identified from 260 systematic reviews

**DOI:** 10.1186/1745-6215-12-122

**Published:** 2011-05-13

**Authors:** Jia He, Liang Du, Guanjian Liu, Jin Fu, Xiangyu He, Jiayun Yu, Lili Shang

**Affiliations:** 1 Chinese Cochrane Centre, Chinese Evidence-Based Medicine Centre, West China Hospital, Sichuan University, Chengdu, 610041, China; 2 West China School of Clinical Medicine, Sichuan University, Chengdu, 610041, China

## Abstract

**Background:**

Randomized controlled trials (RCTs) which are of poor quality tend to exaggerate the effect estimate and lead to wrong or misleading conclusions. The aim of this study is to assess the quality of randomization methods, allocation concealment and blinding within traditional Chinese medicine (TCM) RCTs, discuss issues identified for current TCM RCTs, and provide suggestions for quality improvement.

**Methods:**

We searched Chinese Biomedical Database (CBM, 1978 to July 31, 2009) and the Cochrane Library (Issue 2, 2009) to collect TCM systematic reviews and meta-analyses according to inclusion/exclusion criteria, from which RCTs could be identified. The quality assessment involved whether the randomization methods, allocation concealment and blinding were adequate or not based the study reported. Stratified analyses were conducted of different types of diseases published in different journals (both Chinese and foreign) using different interventions. SPSS 15.0 software was used for statistic analyses.

**Results:**

A total of 3159 RCTs were included, of which 2580 were published in Chinese journals and 579 in foreign journals. There were 381 (12%) RCTs which used adequate randomization methods; 207 (7%) RCTs which used adequate allocation concealment and 601 (19%) which used adequate blinding; there were 130 (4%) RCTs which both used adequate randomization methods and allocation concealment; and there were only 100 (3%) RCTs which used adequate randomization methods, allocation concealment, as well as blinding. In the RCTs published in foreign journals, the adequate randomization methods, allocation concealment and blinding accounted for a relatively large proportion (25%, 26%, and 60%, respectively) and increased with years, while in the RCTs published in Chinese journals, only the adequate randomization methods improved over time. The quality of non-drug intervention (chiefly acupuncture) RCTs was higher than that of drug intervention RCTs. In drug intervention, the quality of listed drugs is higher than the others. The quality of all included RCTs of all types of diseases was generally poor and no studies that were large in size and of high quality were found.

**Conclusion:**

The quality of the current TCM RCTs as judged by their publications is generally poor, especially those published in Chinese journals. In future, researchers of TCM RCTs should attach more importance to experimental design and methodological quality, receive relevant training, and improve reporting quality using the Consolidated Standards of Reporting Trials (CONSORT) statement, so as to improve the quality of TCM clinical research and ensure truth and reliability of conclusions.

## Background

Traditional Chinese medicine (TCM) is centuries old and has developed a unique system of diagnosis and treatment of disease in the practice [1]. TCM is not only an important part of Chinese health but also is accepted in many parts of the world thus offering a complementary or alternative form of health care. International medicine has been increasingly interested in TCM [2-4]. Randomized controlled trials (RCTs) and systematic reviews are commonly regarded first-class evidence in judging the treatment effect of interventions [5,6]. Systematic reviews are based on RCTs, whose deficiency may yield unreliability of the conclusions [7,8], therefore, the quality of included RCTs is of obvious relevance to the interpretability and reliability of the conclusions. Research shows that the RCTs which are of poor quality tend to exaggerate the effect estimates and lead to misleading conclusions [9-16]. The aim of this study is to assess the quality of randomization methods, allocation concealment and blinding of TCM RCTs based on study reports, discuss issues identified for current TCM RCTs, and provide suggestions for quality improvement.

## Methods

### Literature selection

The following databases were searched: Chinese Biomedical Database (CBM, 1978 to July 31, 2009) and the Cochrane Library 2009, Issue 2.

The following search strategies were used:

CBM:

#1 title: systematic review (in Chinese)

#2 keywords: systematic review (in Chinese)

#3 title: systematic overview (in Chinese)

#4 keywords: systematic overview (in Chinese)

#5 title: meta analysis (in Chinese)

#6 keywords: meta analysis (in hinese)

#7 title: meta-analysis (in Chinese)

#8 keywords: meta-analysis (in Chinese)

#9 title: systematic review (in English)

#10 keywords: systematic review (in English)

#11 title: systematic reviews (in English)

#12 keywords: systematic reviews (in English)

#13 title: meta analysis (in English)

#14keywords: meta analysis (in English)

#15 title: meta-analysis (in English)

#16 keywords: meta-analysis (in English)

#17 title: meta analyses (in English)

#18 keywords: meta analyses (in English)

#19 title: meta-analyses (in English)

#20 keywords: meta-analyses (in English)

#21 #1-20/or

The Cochrane Library 2009, Issue 2

#1 herb in Title, Abstract or Keywords

#2 herbal in Title, Abstract or Keywords

#3 herbs in Title, Abstract or Keywords

#4 Chinese in Title, Abstract or Keywords

#5 China in Title, Abstract or Keywords

#6 acupuncture in Title, Abstract or Keywords

#7 alternative complementary medicine in Title, Abstract or Keywords

#8 plant in Title, Abstract or Keywords

#9 moxibustion in Title, Abstract or Keywords

#10 #1~#9/or

All identified RCTs, published in Chinese journals and The Cochrane Library, included in systematic reviews and meta-analyses of TCM (including Chinese herbal medicine, Chinese medicine, Chinese standardized remedies, Chinese medicine preparations and Chinese medicine extracts), integrated TCM and Biomedicine, as well as acupuncture and massage (including acupuncture and electro-acupuncture), were included. Repeatedly-published articles, translated articles, non-full text articles and articles without mentioning sources of RCTs were excluded.

### Quality assessment

The quality assessment in this study was performed according to three major procedures in methodological part of the Cochrane Handbook: ① Whether the randomization method was adequate (such as referring to a randomized number table, using a computer random number generator, coin tossing and throwing dice, etc.) or inadequate (such as sequence generated by odd or even date of birth, sequence generated by some rule based on date of admission or hospital record number, etc.). ② Whether allocation concealment was adequate (such as central allocation, sequentially numbered drug containers of identical appearance, sequentially numbered, opaque, sealed envelopes, etc) or inadequate (such as using an open random allocation schedule, alternation or rotation, date of birth, case record number, etc.). ③ Whether blinding was adequate (such as no blinding, but the review authors judge that the outcome and the outcome measurement are not likely to be influenced by lack of blinding; blinding of participants and key study personnel ensured, and unlikely that the blinding could have been broken; either participants or some key study personnel were not blinded, but outcome assessment was blinded and the non-blinding of others unlikely to introduce bias) or inadequate (such as no blinding or incomplete blinding, and the outcome or outcome measurement is likely to be influenced by lack of blinding; blinding of key study participants and personnel attempted, but likely that the blinding could have been broken, either participants or some key study personnel were not blinded, and the non-blinding of others likely to introduce bias) [17]. Assessment was based on trial publication only. We did not contact trial authors for clarification.

### Stratified analysis

Included RCTs were published in Chinese journals or foreign journals.

Medicine and non-medicine interventions were divided with the former including listed medicines (Chinese standardized remedies, Chinese medicine extracts and single herbs), individualized prescription, mixture (listed medicines and individualized prescription) and unclear (the intervention was not reported).

Types of Diseases: classified according to the 10^th ^version of the international disease classification (ICD-10) [18].

### Data extraction

The researchers used EndNote software to manage titles and abstracts of citations obtained through database search. They(J He, and L Du) read titles and abstracts for primary screening based on the inclusion criteria, and then acquired the full text to read for further screening, before finally deciding whether they would be included or not. Four researchers extracted data, using a pro-forma, and then input the extracted materials into Epidata software. Two other researchers checked the data. Any differences were settled through discussion.

### Data analysis

SPSS 15.0 was used for descriptive analyses and **χ^2 ^**test.

## Results

**Figure 1:**
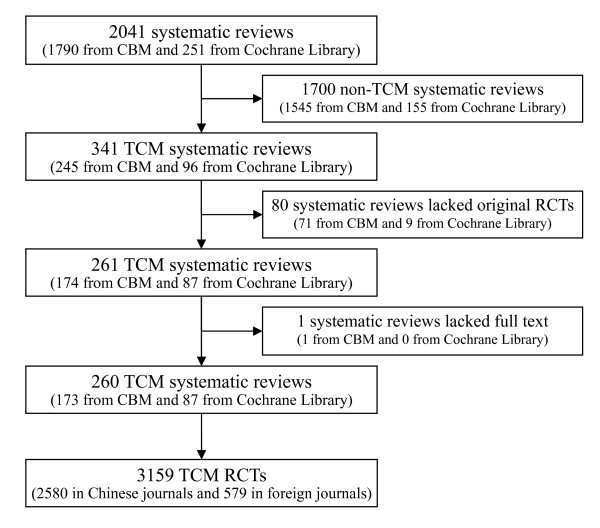
**Flow chart of literature selection**.

A total of 2041 articles were retrieved (1790 from CBM and 251 from The Cochrane Library). After the primary screening, there were 1781 articles were excluded and 260 articles (173 from CBM and 87 from The Cochrane Library) were included. A total of 3159 RCTs were identified from the 260 systematic reviews/meta-analyses, of which 2580 were published in Chinese journals and 579 in foreign journals (Figure 1).

### Assessment of reported randomization methods, allocation concealment and blinding

A total of 3159 RCTs were included, among which 381(12%) used adequate randomization methods; 207 (7%) used adequate allocation concealment; 601 (19%) used adequate blinding; 130 (4%) both used adequate randomization methods and allocation concealment; 100 (3%) were adequate for all three methods.

RCTs published in foreign journals were better than those published in Chinese journals, and the difference was significant (*P = 0.000*) (Table 1).

**Table 1:** Characters of randomization methods, allocation concealment and blinding of included RCTs

Item	Chinese			Foreign			Total			χ2*	P
			
	Adequate	Inadequate	n	Adequate	Inadequate	n	Adequate	Inadequate	N		
	(%)	(%)		(%)	(%)		(%)	(%)			
randomization methods	239(9)	2341(91)	2580	142(25)	437(75)	579	381(12)	2778(88)	3159	103.8	0.000
allocation concealment	56(2)	2524(98)	2580	151(26)	428(74)	579	207(7)	2952(93)	3159	441.4	0.000
blinding	252(10)	2328(90)	2580	349(60)	230(40)	579	601(19)	2558(81)	3159	783.1	0.000
randomization method + allocation concealment	39(2)	2541(98)	2580	91(16)	488(84)	579	130(4)	3029(96)	3159	241.8	0.000
randomization method + allocation concealment + blinding	33(1)	2547(99)	2580	67(12)	512(88)	579	100(3)	3059(97)	3159	163.4	0.000

* Proportion of adequate comparison of Chinese with foreign

### Changes of RCTs reported quality with time

**Figure 2:**
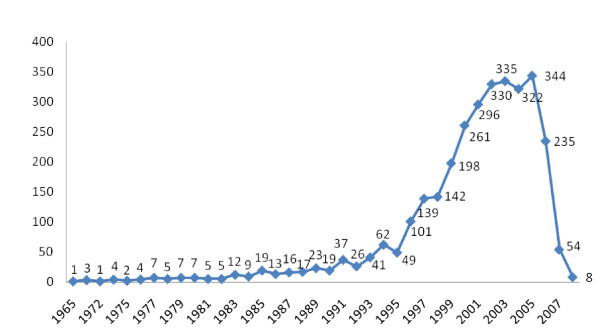
**Numbers of 3159 RCTs with years**.

**Figure 3:**
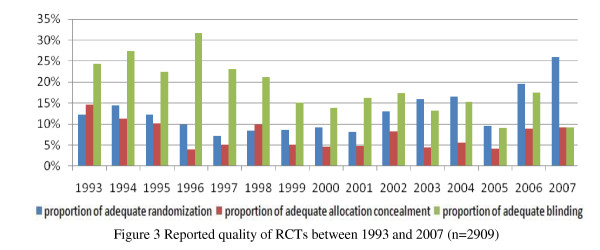
**Reported quality of RCTs between 1993 and 2007 (n = 2909)**.

**Figure 4:**
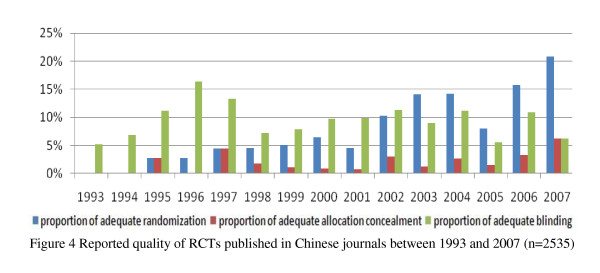
**Reported quality of RCTs published in Chinese journals between 1993 and 2007 (n = 2535)**.

**Figure 5:**
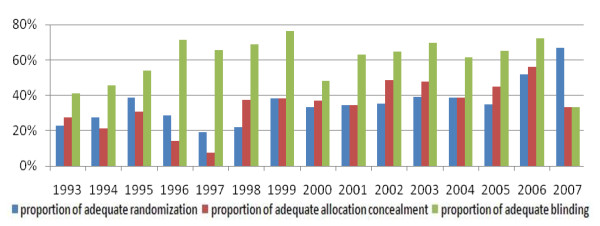
**Reported quality of RCTs published in foreign journals between 1993 and 2007 (n = 374)**.

All 3159 RCTs were published between 1965 and 2008 (Figure 2). The proportion of adequate randomization methods of RCTs increased year by year, but there was no clear increase in allocation concealment and blinding, and the allocation concealment accounted for a very small proportion (Figure 3). In terms of Chinese journals, the proportion of adequate randomization methods of RCTs between 1993 and 2007 increased sharply, but there was no clear increase in the allocation concealment and blinding, and the allocation concealment just occupied a very small proportion (Figure 4). By comparison, there was a large rise in the proportion of adequate randomization methods, allocation concealment and blinding of the RCTs published in foreign journals from 1993 through 2007 (Figure 5). In order to avoid bias caused by chance, RCTs published between 1965 and 1992, as well as in 2008, which number of the included RCTs was very small, were excluded from this analysis.

### Reported quality of RCTs of different interventions

Among 3159 included RCTs, 2546 (81%) were drug interventions and 613 (19%) were non-drug interventions. In the non-drug group, the proportion of adequate randomization methods, allocation concealment and blinding were 21%, 19%, and 38%, respectively, which were larger than those in drug intervention, and the difference were significant (*P = 0.000*) (Table 2).

**Table 2:** Characters of RCTs of different interventions (Chinese journals VS foreign journals)

		Number of RCTs			Adequate randomization methods			Adequate allocation concealment			Adequate blinding		
		
Classification		Chinese	Foreign	Total	Chinese	Foreign	Total	Chinese	Foreign	Total	Chinese	Foreign	Total
					(%)	(%)	(%)	(%)	(%)	(%)	(%)	(%)	(%)
Drug	Chinese standardized remedies	1135	42	1177	140(12)	13(31)	153(13)	32(3)	13(31)	45(4)	148(13)	36(86)	184(16)
	Chinese medicine extracts	241	107	348	7(3)	26(24)	33(9)	3(1)	30(28)	33(9)	12(5)	37(35)	49(14)
	Single herb	100	67	167	3(3)	9(13)	12(7)	0(0)	5(7)	5(3)	5(5)	62(93)	67(40)
	Individualized prescription	562	13	575	41(7)	4(31)	45(8)	1(0)	4(31)	5(1)	24(4)	10(77)	34(6)
	Mixture	115	20	135	4(3)	0(0)	4(3)	0(0)	0(0)	0(0)	10(9)	19(95)	29(21)
	Unclear	141	3	144	4(3)	0(0)	4(3)	3(2)	1(33)	4(3)	3(2)	0(0)	3(2)
	
	Total	2294	252	2546	199(9)	52(21)	**251(10)**	39(2)	53(21)	**92(4)**	202(9)	164(65)	**366(14)**

Non-drug	Acupuncture	263	219	482	37(14)	69(32)	106(22)	17(6)	73(33)	90(19)	49(19)	149(68)	198(41)
	Massage	23	108	131	3(13)	21(19)	24(18)	0(0)	25(23)	25(19)	1(4)	36(33)	37(28)
	
	Total	286	327	613	40(14)	90(28)	**130(21)**	17(6)	98(30)	**115(19)**	50(17)	185(57)	**235(38)**

	***χ2***						*59.99*			*185.10*			*184.11*

	***P***						*0.000*			*0.000*			*0.000*

* Comparison of drugs with non-drugs

In different drug intervention, the proportion of adequate randomization of Chinese standardized remedies RCTs were the largest (13%), the proportion of adequate allocation concealment of Chinese medicine extracts RCTs were the largest (9%), and the proportion of adequate blinding of single herbs RCTs were the largest (40%).

In different non-drug intervention, the proportion of adequate randomization and blinding (22% and 41%, respectively) of acupuncture RCTs were larger, and the proportion of adequate allocation concealment (19%) of acupuncture RCTs was the same with massage RCTs.

In terms of drug intervention RCTs published in foreign journals, except that the proportion of adequate randomization methods of mixed RCTs (including listed medicine and individualized prescription) and unclear RCTs were smaller than those of RCTs published in Chinese journals, other proportion of adequate randomization methods, allocation concealment, and blinding were all larger than those of RCTs published in Chinese journals.

In terms of non-drug RCTs published in foreign journals, the proportion of adequate randomization methods, allocation concealment and blinding were all larger than those of RCTs published in Chinese journals.

### Reported quality of RCTs of different types of diseases

The included 3159 RCTs were classified according to the 10th version of the international disease classification (ICD-10), involving 16 types of diseases and lacking 5 types (Table 3). Quantities and qualities of all types of diseases had imbalanced distribution. Circulatory system RCTs had the largest number but were of low quality.

**Table 3:** Characters of RCTs of different types of diseases (Chinese journals VS foreign journals)

Types of diseases	Number of RCTs			Adequate randomization methods			Adequate allocation concealment			Adequate blinding		
	
	Chinese	Foreign	Total	Chinese	Foreign	Total	Chinese	Foreign	Total	Chinese	Foreign	Total
				(%)	(%)	(%)	(%)	(%)	(%)	(%)	(%)	(%)
Certain infectious and parasitic diseases	197	71	268	7(4)	19(27)	26(10)	6(3)	14(20)	20(7)	13(7)	20(28)	33(12)
Neoplasm	120	33	153	5(4)	1(3)	6(4)	0(0)	14(42)	14(9)	6(5)	12(36)	18(12)
Diseases of the blood and blood-forming organs and certain disorders involving the immune mechanism	32	9	41	1(3)	5(56)	6(15)	1(3)	3(33)	4(10)	2(6)	7(78)	9(22)
Endocrine, nutritional and metabolic diseases	200	6	206	32(16)	0(0)	32(16)	6(3)	0(0)	6(3)	26(13)	4(67)	30(15)
Mental and behavioural disorders	97	96	193	21(22)	12(13)	33(17)	14(14)	8(8)	22(11)	29(30)	85(89)	114(59)
Diseases of the nervous system	56	39	95	4(7)	19(49)	23(24)	0(0)	19(49)	19(20)	2(4)	27(69)	29(31)
Diseases of the eye and adnexa	17	3	20	6(35)	1(33)	7(35)	0(0)	2(67)	2(10)	9(53)	2(67)	11(55)
Diseases of the ear and mastoid process	4	0	4	1(25)	0(0)	1(25)	0(0)	0(0)	0(0)	0(0)	0(0)	0(0)
Diseases of the circulatory system	978	19	997	81(8)	9(47)	90(9)	11(1)	8(42)	19(2)	79(8)	13(68)	92(9)
Diseases of the respiratory system	150	39	189	34(23)	6(15)	40(21)	11(7)	8(21)	19(10)	28(19)	27(69)	55(29)
Diseases of the digestive system	270	14	284	9(3)	5(36)	14(5)	0(0)	8(57)	8(3)	1(0)	7(50)	8(3)
Diseases of the skin and subcutaneous tissue	49	6	55	10(20)	2(33)	12(22)	0(0)	2(33)	2(4)	10(20)	4(67)	14(25)
Diseases of the musculoskeletal system and connective tissue	141	168	309	11(8)	35(21)	46(15)	4(3)	31(18)	35(11)	29(21)	90(54)	119(39)
Diseases of the genitourinary system	220	18	238	11(5)	5(28)	16(7)	3(1)	6(33)	9(4)	16(7)	10(56)	26(11)
Pregnancy, childbirth and the puerperium	42	47	89	6(14)	23(49)	29(33)	0(0)	28(60)	28(31)	2(5)	39(83)	41(46)
Symptoms, signs and abnormal clinical and laboratoty findings, not elsewhere calssified	2	9	11	0(0)	0(0)	0(0)	0(0)	0(0)	0(0)	0(0)	1(11)	1(9)
Factors influencing health status and contact with health services	5	2	7	0(0)	0(0)	0(0)	0(0)	0(0)	0(0)	0(0)	1(50)	1(0)

Total	2580	579	3159	239(9)	142(25)	381(12)	56(2)	151(26)	207(7)	252(10)	349(60)	601(19)

## Discussion

Many studies have showed that RCTs not using randomization, allocation concealment or blinding exaggerate estimates of effect to various extents. Compared with the RCTs using blinding, the RCTs not using blinding yield 17% larger estimates of treatment effects and in trials with subjective outcomes, effect estimates are exaggerated by 25%. Compared with the RCTs using adequate allocation concealment, RCTs using unclear or inadequate concealment of allocation exaggerate estimates of effect by 30%-41% [10-14]. These showed that compared with other "flaws", unclear or inadequate allocation concealment will cause a larger bias, which highlights the importance of allocation concealment. This study indicates that the adequate allocation concealment takes up the smallest proportion (7%) of the three assessed aspects. Although the adequate randomization methods accounted for a larger proportion (12%) than allocation concealment, there are also some investigations which showed that only 6.8% of the RCTs published in Chinese journals were deemed authentic randomized trials [19]. So the quality of the TCM RCTs in this study may be overstated.

Compared with those published in Chinese journals, the TCM RCTs published in foreign journals are of higher quality. In terms of changes over time, the proportion of reports of adequate randomization has increased year on year, suggesting that more attention has been paid to the randomization methods of Chinese TCM RCTs. However, the proportion of adequate allocation concealment and blinding are small and has remained at approximately the same level. By contrast, the proportion of adequate randomization methods, allocation concealment and blinding of the TCM RCTs published in foreign journals has increased overtime. This suggests that Chinese journals are failing to improve the standards of published RCTs.

Acupuncture, thousands of years of history in China, is a traditional medicine recognized by the World Health Organisation and has become a component of health care in many countries [20]. Since late 1990s, numbers of acupuncture clinical trials have grown dramatically with the increase of research funds in western countries [21]. The results of this study shows that in the light of interventions of TCM RCTs, the quality of non-drug intervention is evidently higher than that of drug intervention, and acupuncture is the largest group. In drug intervention, the quality of RCTs of Chinese standardized remedies, Chinese medicine extracts and single herbs is comparatively high while that of individualized prescription, mixtures (including listed medicine and individualized prescription) and those where the intervention is unclear is low. Individualized prescription which is common in TCM is influenced by a doctor's experience and training. There are no agreed formulae for drug ingredients, no standard sources of drugs, or preparation methods. In addition, doses of Chinese herbs are not standard. Listed medicine and single herbs can overcome the above shortcomings of individualized prescription, so the quality of RCTs of these kinds is higher and clinical research develops in a more extensive way.

With the development of TCM, current TCM RCTs involve wide study fields and many types of diseases. This study was aimed at determining which medical specialties produce with high-quality RCTs, but these were not identified.

The quality assessment in this study was based on how the original study was reported, so it was affected by the quality of reporting. There also may be some other factors not in the power of the reseachers such as journal word length etc. Some studies have showed that use of the Consolidated Standards of Reporting Trials (CONSORT) statement is associated with improvements in the reporting quality of RCTs, but few researchers and journals adopt it [22-30]. The CONSORT extensions for acupuncture (the Standards for Reporting Interventions in Clinical Trials of Acupuncuture, STRICTA) and herbal medicine were designed to improve the completeness and transparency of reporting of interventions in controlled trials of acupunture and herbal medicine [31,32].

## Conclusion

The quality of the current TCM RCTs as judged by their publications is generally poor, especially those published in Chinese journals. In future, researchers should attach more importance to design and methodological quality, especially to allocation concealment. The quality of RCTs can also be improved through promoting participation of methodologists and statisticians, enhancing international cooperation, and adopting guidelines such as the CONSORT statement and its extensions for acupuncture and herbal medicine. Meanwhile, medical journals should also be more discerning in what they accept for publication, so as to improve the quality of TCM clinical research and ensure truth and reliability of conclusions.

## Abbreviations

RCT: randomized control trial; TCM: traditional Chineses medicine; CBM: Chinese Biomedical Database; CONSORT: Consolidated Standards of Reporting Trials; STRICTA: Standards for Reporting Interventions in Clinical Trials of Acupuncuture.

## Competing interests

The authors declare that they have no competing interests.

## Authors' contributions

JH and LD participated in the design of the study and drafted the article. GL conceived the study, interpreted the data and revised the manuscript. JF, XH, JY and LS extracted the data and performed the statistical anlysis. All authors read and approved the manuscript.
